# The supervisory relationship as a predictor of mental health outcomes in doctoral students in the United Kingdom

**DOI:** 10.3389/fpsyg.2024.1437819

**Published:** 2024-10-09

**Authors:** Alkistis P. Mavrogalou-Foti, Maria A. Kambouri, Soljana Çili

**Affiliations:** ^1^Department of Psychology and Human Development, Institute of Education, University College London, London, United Kingdom; ^2^London College of Fashion, University of the Arts London, London, United Kingdom

**Keywords:** doctoral students, mental health, supervisory relationship, postgraduate studies, supervision

## Abstract

**Introduction:**

The process of a doctorate degree has been implicated in the onset and exacerbation of mental health problems among doctoral students. Previous studies have suggested that the student-supervisor relationship may predict emotional wellbeing and mental health outcomes in doctoral students in the UK. However, these studies were conducted before the COVID-19 pandemic and often used unstandardized measures to investigate supervisory styles.

**Methods:**

The present study was part of the Better Together project, a wellbeing initiative for doctoral students in the UK. It explored the predictive ability of aspects of the student-supervisor relationship with regards to doctoral students’ mental health outcomes. The sample consisted of 141 students doing a research-based doctorate degree in the UK. The survey included demographic questions and questionnaires assessing supervisory styles, the discrepancy between actual and preferred supervisory relationship, depression, anxiety, and stress.

**Results:**

A large proportion of participants fell in the severe and extremely severe categories in the depression, anxiety, and stress sub-scales. Multiple regression analyses indicated that both supervisory styles and discrepancy significantly predicted students’ mental health outcomes. More specifically, higher scores in the uncertain supervisory style, which is characterized by indecisiveness and ambiguity, were linked with higher scores in depression, anxiety, and stress.

**Discussion:**

The findings provided new insights concerning the aspects of the student-supervisor relationship that are related to the mental health issues of doctoral students in the UK. They have important implications for future research and supervision practice.

## 1 Introduction

Over the last years, there has been an increase in the total number of individuals pursuing a doctorate degree in the United Kingdom (UK; HESA, 2023). The process of a doctorate is both intellectually and emotionally challenging, with students often expressing concerns about work-life balance, supervisory relationships, financial pressures, and social isolation (Metcalfe et al., 2018). Recently, research has focused on the effects of pursuing a doctorate degree on doctoral students’ mental health and wellbeing, as well as on individual, social, and/or occupational factors that could be associated with these effects. This is a result of a general concern over individuals’ wellbeing and the considerable cost to research institutions and teams (see Podsakoff et al., 2007). The aim of this paper is to explore the prevalence of mental health issues in doctoral students in the UK and investigate whether it is associated with aspects of the student-supervisor relationship.

### 1.1 Mental health concerns

The doctorate process has been implicated in the onset and exacerbation of mental health problems and reduction of wellbeing (Berry et al., 2020; Friedrich et al., 2023; Levecque et al., 2017). This phenomenon has been described as a mental health crisis (Evans et al., 2018) since a substantial proportion of doctoral students has been found to experience clinically relevant mental health symptoms, including depression, anxiety, and stress. For example, Levecque et al. (2017) compared doctoral students in Belgium to three other age-matched groups, including highly educated people in the general population, higher education students, and highly educated employees. They found that doctoral students were significantly more likely to be at risk of having or developing a common psychiatric disorder, with a particularly high risk for depression. Approximately 32% of the doctoral students in the study reported psychological distress. Distress was subjectively measured, with authors deciding on the minimum number of mental health symptoms above which participants were considered as having psychological distress. Hazell et al. (2021) found that UK doctoral students report significantly greater clinically relevant (mild to severe) symptoms of depression and anxiety compared to educated age-matched control groups, even after controlling for pre-existing mental health problems. Similar results have been replicated in North America (e.g., Evans et al., 2018) and Australia (e.g., Barry et al., 2018). Doctoral students have also been found to report higher levels of stress compared to the general population (Barry et al., 2018; Hazell et al., 2021).

The recent COVID-19 pandemic resulted in a further increase in mental health problems and poor wellbeing in the general population of the UK (e.g., O’Connor et al., 2021; Zavlis et al., 2021) and in doctoral students specifically (e.g., Byrom, 2020; Sideropoulos et al., 2022). A qualitative study (Jackman et al., 2022) explored the effects of lockdown and the pandemic in doctoral students and early career researchers in the UK, with participants reporting increased stress and anxiety, as well as reduced wellbeing. Moreover, a cross-sectional study (Byrom, 2020) indicated that the majority of doctoral students and early career researchers reported some level of mental distress and low levels of mental wellbeing at the beginning of the pandemic. Both these studies included doctoral students and university research staff together, limiting our understanding of the pandemic experiences of doctoral students specifically.

Even though there has been an increase in policy makers’ and institutions’ understanding of the mental health crisis, doctoral students often do not access institutional support and instead prefer turning to external support mechanisms including family, peers, and online resources (Berry et al., 2020; Metcalfe et al., 2018; Waight and Giordano, 2018). Partly because of this, in recent years there has been an increase in studies which investigate the factors and experiences affecting doctoral students in order to potentially create intervention and/or prevention programs within institutions to support these students’ psychological wellbeing. Among the experiences that have been studied is the nature of the relationship that doctoral students have with their supervisors.

### 1.2 Supervisory relationship

The supervisor is one of the most important sources of support for doctoral students and has therefore received considerable attention in research (Sverdlik et al., 2018). The supervisor’s role is complex as it involves both an intellectual dimension including knowledge around a topic, feedback, and guidance, and an affective dimension including support and friendliness (Halse and Malfroy, 2010). Gurr (2001) proposes that the supervisor promotes both the progress of the doctoral research and the doctoral student’s overall development as a researcher. These require sensitivity and flexibility toward the student’s needs (Gurr, 2001).

Several studies have explored what a good and supportive supervision includes from the perspective of the student (e.g., Halbert, 2015), the supervisor (e.g., Bengtsen and McAlpine, 2022), and from both students’ and supervisors’ perspective (e.g., Moxham et al., 2013). A good and supportive supervision has been linked with less emotional exhaustion (Hunter and Devine, 2016) and has been associated with frequent meetings, open discussion, encouragement, and precise and timely feedback (Latona and Browne, 2001, as cited in Sverdlik et al., 2018). Moxham et al. (2013) suggest that both supervisor and supervisee acknowledge the importance of their relationship and the need for open communication about each party’s expectations from that relationship and from the doctorate journey. Additionally, Halbert (2015) suggests that doctoral students value a supervisor who is supportive, personal, flexible, and responsive. The study identified several important aspects of the supervisory relationship, such as appropriate feedback and knowledge of the field and research process, with the majority of participants focusing on the supervisors’ interpersonal characteristics as a key determinant of a good and quality supervision. Among other things, they emphasized consistent and regular contact, as well as the supervisor being approachable, respectful, and understanding.

The existing literature presents divergent views about what makes supervision good and supportive. This is likely the result of studies originating from different countries and evaluating the doctorate journey of different disciplines, where the doctorate degree and the role of the supervisor vary. For example, in STEM disciplines doctoral students usually work in research groups and supervision is a group process rather than a supervisor-student dyad (e.g., Chiang, 2003). Despite these differences, there seems to be a consensus that supervision requires good interpersonal communication.

### 1.3 Interpersonal model of supervision

The interpersonal model of supervision is a framework used to describe the student-supervisor relationship (Mainhard et al., 2009). It was first developed to analyze teacher-student interactions in secondary classrooms (Wubbels et al., 2006). The model emphasizes that the longer a student and supervisor interact and communicate, the more predictable their interactions become, and mutual expectations slowly develop (Wubbels et al., 2006). These patterns overall form certain expectations of behavior, referred to as interpersonal styles of behavior. These styles depend both on the supervisor and the student, and supervisors might display different behaviors when interacting with different students (Mainhard et al., 2009).

**Figure 1:**
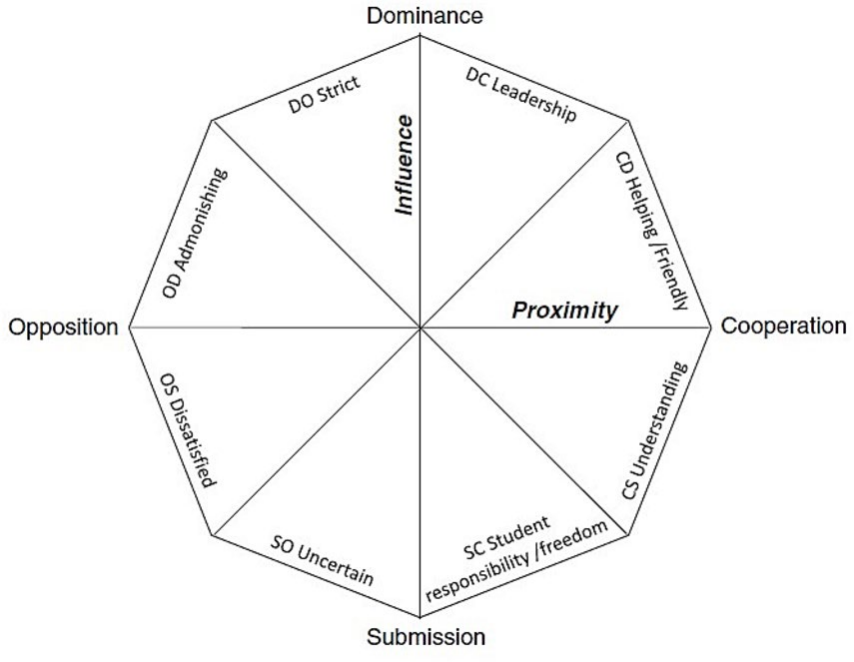
The interpersonal model of supervision. Reproduced with permission of Springer Nature from “A Model for The Supervisor-Doctoral Student Relationship,” by Mainhard et al. (2009), p. 363.

Based on this model, the supervisory relationship is characterized by the two independent dimensions of Influence (Dominance—Submission) and Proximity (Opposition—Cooperation), which are believed to be the universal descriptors of human interaction (Wubbels et al., 2006). The two dimensions are represented in two axes and overall contain eight types of behaviors that the supervisor might display (Figure 1). These supervisory styles include leadership (e.g., giving guidance, being responsive), helpfulness/friendliness (e.g., supportive, cooperative), understanding (e.g., trusting and pays attention), giving PhD student responsibility/freedom (e.g., accepting student’s proposals and decisions), uncertain (e.g., indecisiveness and ambiguity), dissatisfied (e.g., dissatisfied about progress), admonishing (e.g., impatient and bad tempered), and strict (e.g., critical and demanding; Mainhard et al., 2009).

Behaviors closer to the Proximity axis (i.e., helping/friendly, understanding, dissatisfied, admonishing) contribute more to the Proximity dimension and reversely, behaviors closest to the Influence axis (i.e., leadership, student responsibility/freedom, uncertain, and strict) contribute more to the Influence dimension. This is similar to another model of supervision which identifies four styles of supervision, based on the dimensions of structure and support, including directional (good guidance but non-supportive), *laissez-faire* (non-supportive and uninvolved), contractual (both support and guidance), and pastoral (high support but little guidance) supervisory style (Gatfield, 2005). However, in the interpersonal model, a supervisor has a distinct degree of intensity for each supervisory style and not just a single supervisory style. For example, a supervisor might simultaneously display a behavior closer to the center, indicating low intensity of that supervisory style, and a behavior closer to the endpoint of the axis, indicating high intensity. Arguably, this provides a richer understanding of supervision, as supervisory styles are mapped as different degrees of behavior intensity instead of just referring to the presence of a single supervisory style.

Supervisors’ interpersonal style of behavior may be explored from the perspective of doctoral students using the Questionnaire of Supervisor-Doctoral student Interaction (QSDI; Mainhard et al., 2009). This questionnaire includes 41 statements of possible behaviors that the supervisor might display, and the student has to rate them on a scale of *never/not at all* to *always/very*. These statements are then grouped into 8 degrees of supervisory styles. It is important to note that the intercorrelation between the eight types of behavior could create ambiguity when trying to identify the effects of different supervisory styles on doctoral students as it does not fully distinguish between different types of supervision. However, Mainhard et al. (2009) highlight that there is not one single aspect of a supervisor’s style that defines their supervision, and their questionnaire is one of the most widely cited explanations of supervisory styles.

### 1.4 ‘Fit’ of supervision: preferred and actual supervision

Another aspect of the student-supervisor relationship that has been briefly discussed in the literature is “fit” of supervision, that is whether doctoral students and supervisors have similar perceptions and expectations from each other. Deuchar (2008) suggests that the supervision provided to doctoral students is not always the type of guidance and support that they are looking for. Sometimes, for example, doctoral students need academic support whereas supervisors offer them pastoral support (Deuchar, 2008). This could create unmet expectations in students, as well as a discrepancy between what the students receive and what they need at a certain point in time. Additionally, it has been suggested that good and supportive supervision needs to be tailored to fit the needs of each student (Watts, 2008) and that these needs might change throughout the doctorate journey (Gurr, 2001). In the interpersonal model of supervision, Mainhard et al. (2009) argue that it is very important to explore the preferred and actual experienced supervisory interactions of doctoral students and any potential discrepancy between the two. This may help us understand doctoral students’ need for a specific supervisory style and identify ways in which the supervisory relationship can be improved.

### 1.5 Supervisory relationship and mental health outcomes

Research on the prevalence of mental health issues in doctoral students (e.g., Barry et al., 2018) suggests that a good and supportive supervision, as well as the communication between student and supervisor, is vital for students’ experience and overall emotional wellbeing. Levecque et al. (2017) found that the leadership supervisory style is linked to mental health outcomes in doctoral students. More specifically, an inspirational leadership was negatively associated with psychological distress and risk of experiencing a common psychiatric disorder (i.e., anxiety, depression, social withdrawal, and somatic symptoms), whereas a *laissez-faire* leadership style was positively associated with psychological distress. However, these authors did not use a standardized measure for supervision. They do not explain how supervisory styles were examined and what each style entails. Corner et al. (2017) have also suggested associations between reduced levels of satisfaction with the supervisory relationship and higher reports of stress and emotional exhaustion in doctoral students. A qualitative study conducted in the UK indicated that the relationship of doctoral students with their supervisor is often perceived as asymmetrical, with doctoral students suggesting that supervisors need to be better equipped to support students’ mental health and emotional wellbeing (Berry et al., 2020).

When it comes to “fit” of supervision, several empirical studies suggest that it is significantly related to the emotional wellbeing of doctoral students (e.g., McAlpine and McKinnon, 2013). A qualitative study by Cotterall (2013) examined a small sample of doctoral students for 2 years to explore the most commonly occurring emotion-eliciting elements along their doctorate journey. Results indicated that supervision was mostly described as positive, with descriptions of good feedback, communication, and support. However, the discrepancy between supervisors’ and students’ expectations was a phenomenon that caused confusion, stress, and anxiety in several doctoral students. Similarly, Sverdlik et al. (2018) suggest that the fit of supervision affects doctoral students’ emotions and persistence in the doctorate journey. Despite these findings, to date there has not been an investigation of the direct associations between the discrepancy of actual and preferred supervision and mental health outcomes.

A growing body of quantitative research investigates doctoral students’ mental health and possible contributing factors. These factors include individual (e.g., age), interpersonal (e.g., social support) and institutional (e.g., discipline) characteristics that all seem to interact and influence students’ mental health issues (Casey et al., 2023; Milicev et al., 2021; Sverdlik et al., 2018). This interplay of factors has created a debate whereby some authors question the relevance and importance of supervision for the mental health of doctoral students as other factors show greater associations with it. For example, academic challenges, including managing time and other work commitments, have been singled out as the factor with the greatest negative impact on doctoral students’ wellbeing (e.g., Crook et al., 2021; Milicev et al., 2021).

In the context of the UK, a pre-pandemic study evaluated the mental health of doctoral students and indicated that a large percentage of participants reported severe symptoms of anxiety and depression (20 and 23%, respectively; Milicev et al., 2021). The authors also investigated whether individual (e.g., age, sex) and environmental factors (e.g., self-reported evaluations of progress and supervisory relationship) could predict participants’ mental health issues. They found that maladaptive perfectionism and workaholism were linked to negative outcomes for mental health, whereas resilience, adaptive perfectionism, and supervisory relationship were linked to positive outcomes, with supervisory relationship indicating a smaller predictive ability (Milicev et al., 2021). The authors did not use an existing standardized measure of supervisory relationship. They created a novel instrument and only seemed to distinguish between good and bad supervision. This fails to acknowledge the complexity of the supervisory relationship.

Interestingly, a longitudinal study by Gooding et al. (2023) indicated that only the perception of academic challenges at Time 1 predicted anxiety at Time 2 relative to baseline. The authors did not find any association between perceptions of supervisors and mental health problems or wellbeing over time. However, they acknowledge that the supervisory relationship was subjectively measured using the item “To what extent has your supervisor negatively affected your wellbeing?” and that a more in-depth examination of the supervisory relationship (e.g., in terms of the quality of supervisors’ academic input) is required.

Another pre-pandemic study investigated the predictive ability of a set of factors on depression, anxiety, and suicidality using the QSDI to explore the supervisory relationship. Berry et al. (2021) combined the items of the scale into two dimensions of agency (influence and leadership), and communion (proximity and cooperativeness). Using hierarchical regression, they found that low communion was associated with symptoms of depression and anxiety, whereas agency only predicted depression. Additionally, a follow-up study (Berry et al., 2023) indicated that lower agency was related to lower attendance behaviors, measured using binary categorical variables of absenteeism (days absent) and presenteeism (working days that were affected by physical or psychological problems) in the past month, excluding planned annual leave or holidays. At the same time, lower communion predicted mental-health-related attrition intention. These studies are important as they relied on the interpersonal model of supervision. However, they reduced the supervisory styles into two dimensions. Given that each style has a certain degree of proximity and influence (Mainhard et al., 2009), it is unknown whether the two dimensions actually indicate a measure of proximity and influence independently. Additionally, both studies tested hierarchical logistic regression models with a large number of variables and thus the individual contribution of each variable is difficult to interpret.

### 1.6 Present study

Although it is evident from the above that there are a substantial number of studies exploring the prevalence of mental health issues as well as the associations between mental health issues and factors influencing the doctorate journey, they present significant limitations. The majority do not explore the supervisory relationship through a robust theoretical framework and use unvalidated standardized methods for assessing it. Further studies are needed in order to understand the predictive ability of the supervisory relationship in terms of doctoral students’ mental health issues using a model of supervision which recognizes the nuances of different supervisory styles. This could provide a more holistic understanding of students’ experience and needs throughout their doctorate journey.

The present study investigated the predictive ability of aspects of the student-supervisor relationship with regards to doctoral students’ mental health outcomes, specifically depression, anxiety, and stress. A more thorough investigation of supervisory styles could shed light on more specific aspects of the relationship that might influence doctoral students’ mental health following the challenges posed by the pandemic. This study, therefore, aimed to fill a gap in the existing literature on UK-based doctoral students. It was part of the Better Together project, a wellbeing initiative at University College London (UCL) which aimed to explore the student-supervisor relationship from the perspective of the doctoral student and investigate whether this relationship can predict educational and mental health outcomes in doctoral students. Using a cross-sectional design, the study aimed to answer two research questions: (1) Which supervisory styles predict students’ depression, anxiety, and stress? (2) Is the discrepancy between actual and preferred supervisory relationship related to students’ depression, anxiety, and stress? Based on the existing literature, it was hypothesized that specific supervisory styles, as well as the discrepancy between actual and preferred supervisory relationship, would predict students’ levels of depression, anxiety, and stress.

## 2 Methods

### 2.1 Participants

The participants were a convenience sample of doctoral students doing a research-based degree in a UK university. They were recruited through student lists, word-of-mouth, and emails sent to supervisors across UK universities which were then forwarded to their doctoral students. As the aim of the study was to investigate the importance of the supervisor for doctoral students’ mental health and research self-efficacy (i.e., perceived ability to perform research-related tasks; Bieschke et al., 1996), only students doing a research-based degree were included. Doctoral students doing a professional or practice-based doctorate were excluded from the study because their research is only one component of the doctorate degree, and it is therefore possible that supervisors play a different role.

In total, 187 doctoral students attempted to complete the study. However, 42 responses were excluded either because the participants did not complete all the questionnaires or because they did not meet the inclusion criteria. The final sample consisted of 141 doctoral students (*M*_Age_ = 31.21 years, *SD* = 8.01). Table 1 presents participants’ personal and occupational characteristics.

**Table 1:** Demographic and occupational characteristics by number of participants (n) and percentage (%).

Characteristic	*n*	%
Sex		
Male	39	27.7
Female	97	68.8
Prefer not to say	5	3.5
Ethnicity		
White British	66	46.8
White Other	32	22.7
Chinese/Chinese British	13	9.2
Black/African/Caribbean/Black British	6	4.3
Asian/Asian British	8	5.7
Mixed ethnicity	3	2.1
Other ethnicity	10	7.1
Prefer not to say	3	2.1
Part of research group		
Yes	72	51.8
No	67	48.2
Funding		
Self-funded	28	20.0
Partially funded	4	2.9
Fully funded	108	77.1
Mode of study		
Full-time	127	90.7
Part-time	13	9.3

### 2.2 Materials

#### 2.2.1 Student-supervisor relationship

The QSDI (Mainhard et al., 2009) is a 41-item questionnaire designed to explore the student-supervisor relationship, from the perspective of the student, based on their interpersonal style of communication. The questionnaire assesses eight types of behaviors: leadership, helping/friendly, understanding, giving PhD student responsibility/freedom, uncertain, dissatisfied, admonishing, and strict. Each item begins with the phrase *“My supervisor….”* For example, the item *“is uncertain during our meetings”* is included in the uncertain supervisory style, the item *“gives me clear guidance”* is included in the leadership supervisory style, while the item *“has a bad temper during our meetings”* is included in the admonishing supervisory style. Items are scored on a 5-point Likert scale ranging from 1 *(“Never/Not at all”)* to 5 *(“Always/Very”)*. The average score of the items that fall under each style is calculated and indicates the extent to which supervisors engage in or exhibit each supervisory style according to the students. In this study, Cronbach’s alpha for the eight supervisory styles ranged from 0.70 to 0.95.

#### 2.2.2 Actual-preferred supervisory relationship discrepancy scale

The Self-Discrepancy Index (e.g., Dittmar et al., 1996) was adapted to explore whether doctoral students have different perceptions of how their relationship with their supervisor should be. In the Self-Discrepancy Index, participants are asked to complete the sentence *“I am… but I would like….”* Consequently, they are asked to rate the magnitude of the discrepancy (“how different” their actual self is from their ideal self) and its salience (“how concerned” they are about that difference) on a scale from 1 *(“A little”)* to 6 *(“Extremely”)*. For the purposes of this study, we created the Actual-Preferred Supervisory Relationship Discrepancy Scale, in which participants had to complete the sentence *“My relationship with my PhD supervisor is… but I would like it to be…”* and rate it on a scale from 1 to 5. The discrepancy index is calculated by multiplying the magnitude and salience scores (Dittmar et al., 1996). Thus, in our study values ranged from 1 to 25, with 25 indicating the highest discrepancy between actual and preferred supervisory relationship.

#### 2.2.3 Mental health

The Depression, Anxiety, Stress Scale (DASS-21; Lovibond and Lovibond, 1995) is a 21-item scale used to measure the negative emotional states of depression, anxiety, and stress. For example, the item *“I found myself agitated”* is included in the stress sub-scale while the item *“I found it difficult to work up the initiative to do things”* is included in the depression sub-scale. Participants rate each statement on a scale from 0 *(“Did not apply to me at all”)* to 3 *(“Applied to me very much or most of the time”)*, indicating how much they have experienced each state over the past month. The scores of each sub-scale are calculated by summing each item of the sub-scale and multiplying by 2. Therefore, scores range from 0 to 42, with a value of 42 indicating that the person is in the extremely severe category of that scale. In this study, the Cronbach’s alpha values of each sub-scale ranged from 0.81 to 0.89, indicating high internal consistency.

### 2.3 Procedure

The study received ethical approval from the UCL Institute of Education. The data were collected online using Qualtrics. Prior to data collection, five preliminary semi-structured interviews were conducted with doctoral students in order to thoroughly understand their perspective of the student-supervisor relationship and to make sure that our questionnaires included all relevant areas. The semi-structured interviews were not recorded, and the participants first replied to a set of questions and then completed the questionnaires. After the interviews, minor changes were made. For example, the QSDI and the Actual-Preferred Supervisory Relationship Discrepancy Scale were rephrased in order to refer to the first supervisor as most doctoral students seemed to have two supervisors, with the first one being closer to them.

An information sheet preceded the questionnaires that informed participants on the aims, procedure, inclusion criteria, and associated risks of the study. Informed consent was sought prior to testing. Subsequently, students answered a series of demographic questions and questions related to their PhD. They also completed the QSDI, Actual-Preferred Supervisory Relationship Discrepancy Scale, DASS-21, and the Research Self-Efficacy Scale (RSES, Bieschke et al., 1996). The RSES was used in a different analysis, not included in this paper, to explore associations between research self-efficacy and supervisory relationship. A debrief form was provided at the end of the survey. The survey took approximately 20–25 min to complete, and participants had the right to withdraw at any point during the study.

### 2.4 Data analysis

All analyses were conducted using SPSS version 28.0. Descriptive statistics were initially obtained and then six multiple regression analyses were conducted. Prior to the analyses, initial statistical tests were conducted in order to check for the assumptions of multiple regression. The understanding supervisory style was strongly correlated with the other supervisory styles (*r* = 0.77 with leadership, *r* = 0.86 with helping/friendly, *r* = 0.84 with PhD student responsibility/freedom, *r* = −0.66 with uncertain, *r* = −0.75 with dissatisfied, and *r* = −0.75 with admonishing) and the assumption of multicollinearity was violated. To address this issue, we removed the understanding supervisory style from the analysis. Its potential overlap with the other styles could have meant that this variable was redundant. Although variable deletion may introduce a certain degree of bias, it is in line with recommendations from Field (2018), Sprinthall (2014), and Wooldridge (2020). The first three multiple linear regressions used the QSDI supervisory styles as predictors of depression (Model 1), anxiety (Model 2), and stress (Model 3). Then, the Actual-Preferred Supervisory Relationship Discrepancy Scale was used as a predictor of depression (Model 4), anxiety (Model 5), and stress (Model 6). Additionally, content analysis was performed in the open-ended questions of the Actual-Preferred Supervisory Relationship Discrepancy Scale to get a deeper and more thorough understanding of the perceived discrepancy between students’ actual experience and expectations of the supervisory relationship.

## 3 Results

### 3.1 Descriptive statistics for DASS-21

A high proportion of the sample reached the severe and extremely severe categories of depression, anxiety, and stress. Table 2 presents the number and proportion of participants under each category.

**Table 2:** Number (n) and percentage (%) of participants under each category of DASS-21.

Category	*n*	%
Depression		
Normal	59	41.8
Mild	24	17.1
Moderate	27	19.1
Severe	12	8.5
Extremely Severe	19	13.5
Anxiety		
Normal	54	38.3
Mild	18	12.8
Moderate	23	16.3
Severe	17	12.0
Extremely Severe	29	20.6
Stress		
Normal	52	36.9
Mild	17	12.0
Moderate	34	24.1
Severe	20	14.2
Extremely Severe	18	12.8

### 3.2 Supervisory styles and mental health outcomes

In the first three multiple regression analyses that were conducted (see Table 3 for coefficients, Table 4 for model outputs), the supervisory styles explained 26% of the variance on depression scores, 12% of the variance on anxiety scores, and 23% on stress scores. The uncertain supervisory style was the only significant predictor on the sub-scales of depression (*β* = 5.36, *p* = 0.011), anxiety (*β* = 5.68, *p* = 0.004), and stress (*β* = 8.57, *p* < 0.001). That means that for each unit increase on the uncertain supervisory style, there is approximately a five-unit change on depression and anxiety scores and an eight-unit change on stress scores. For all mental health outcomes, that is approximately a change in category (e.g., from normal to mild symptoms).

**Table 3:** Coefficients of mental health outcomes.

Model		Unstandardized	Standard error	Standardized	*t*	*p*
Model 1—Depression and QSDI	(Intercept)	4.34	8.63		0.50	0.62
	Leadership	2.87	2.00	0.25	1.44	0.15
	Helping/Friendly	−2.50	2.14	−0.21	−1.17	0.25
	PhD student responsibility/freedom	−1.17	1.63	−0.09	−0.72	0.47
	**Uncertain**	**5.36**	**2.07**	**0.34**	**2.60**	**0.01**
	Dissatisfied	0.62	2.70	0.04	0.23	0.82
	Admonishing	1.88	2.18	0.14	0.86	0.39
	Strict	−0.41	1.35	−0.03	−0.30	0.76
Model 2—Anxiety and QSDI	(Intercept)	1.01	8.02		0.13	0.90
	Leadership	1.86	1.86	0.19	1.00	0.32
	Helping/Friendly	0.26	1.99	0.03	0.13	0.90
	PhD student responsibility/freedom	−1.86	1.52	−0.17	−1.23	0.22
	**Uncertain**	**5.68**	**1.92**	**0.42**	**2.96**	**0.004**
	Dissatisfied	0.61	2.51	0.05	0.24	0.81
	Admonishing	−1.16	2.03	−0.10	−0.57	0.57
	Strict	−0.31	1.26	−0.03	−0.25	0.80
Model 3—Stress and QSDI	(Intercept)	5.69	9.05		0.63	0.53
	Leadership	2.08	2.10	0.18	0.99	0.32
	Helping/Friendly	1.17	2.25	0.10	0.52	0.60
	PhD student responsibility/freedom	−2.84	1.71	−0.22	−1.65	0.10
	**Uncertain**	**8.57**	**2.17**	**0.53**	**3.95**	**<0.001**
	Dissatisfied	−3.16	2.83	−0.20	−1.12	0.27
	Admonishing	1.79	2.29	0.14	0.78	0.44
	Strict	−0.83	1.42	−0.06	−0.58	0.56

Bold values indicate the significant (*p* ≤ 0.01) predictors of each model.

**Table 4:** Multiple linear regression outputs.

Model	Output
Model 1—Depression and QSDI	*F*(7, 126) = 6.42, *p* < 0.001, *R*^2^ = 0.26, *R*^2^ adjusted = 0.22
Model 2—Anxiety and QSDI	*F*(7, 126) = 2.52, *p* = 0.019, *R*^2^ = 0.12, *R*^2^ adjusted = 0.074
Model 3—Stress and QSDI	*F*(7, 126) = 4.23, *p* < 0.001, *R*^2^ = 0.23, *R*^2^ adjusted = 0.22
Model 4—Depression and Discrepancy Scale	*F*(1, 133) = 38.84, *p* < 0.001, *R*^2^ = 0.19, *R*^2^ adjusted = 0.15
Model 5—Anxiety and Discrepancy Scale	*F*(1, 132) = 7.22, *p* = 0.008, *R*^2^ = 0.052, *R*^2^ adjusted = 0.045
Model 6—Stress and Discrepancy Scale	*F*(1, 133) = 9.87, *p* = 0.002, *R*^2^ = 0.069, *R*^2^ adjusted = 0.062

### 3.3 Actual-preferred supervisory relationship discrepancy scale and mental health outcomes

In the exploratory multiple regression analyses that were conducted, the Actual-Preferred Supervisory Discrepancy Scale significantly predicted scores on the sub-scales of depression (*β* = 0.77, *p* < 0.001), anxiety (*β* = 0.32, *p* = 0.008), and stress (*β* = 0.43, *p* = 0.002; see Table 5 for coefficients, Table 4 for model outputs). The scores on the discrepancy scale explained 19% of the variance on depression, 5.2% of the variance on anxiety, and 6.9% on stress scores.

**Table 5:** Coefficients of mental health outcomes.

Model		Unstandardised	Standard error	Standardised	*t*	*p*
Model 4—Depression and Discrepancy Scale	(Intercept)	9.46	1.01		9.32	<0.001
	**Discrepancy Scale**	**0.77**	**0.12**	**0.48**	**6.23**	**<0.001**
Model 5—Anxiety and Discrepancy Scale	(Intercept)	9.32	0.96		9.71	<0.001
	**Discrepancy Scale**	**0.32**	**0.12**	**0.23**	**2.69**	**0.008**
Model 6—Stress and Discrepancy Scale	(Intercept)	16.73	1.11		15.01	<0.001
	**Discrepancy Scale**	**0.43**	**0.14**	**0.26**	**3.14**	**0.002**

Bold values indicate the significant (*p* ≤ 0.01) predictors of each model.

### 3.4 Content analysis

When describing their current (actual) relationship with their supervisor *(“My relationship with my PhD supervisor is…”)*, most participants reported positive characteristics (e.g., supportive, professional, friendly, reliable). Others highlighted negative aspects of the supervisor (e.g., fearful, uncertain, anxiety- or stress-provoking, and diminishing). When asked to describe how they would ideally like their supervisory relationship to be, participants tended to refer to behaviors that they would like to see more from their supervisor. The majority indicated that they would like their supervisor to be more friendly/informal, supportive, straight-forward, organized, and available for more frequent meetings between them. Other participants referred to behaviors that they would like to see less, such as their supervisor being less anxiety provoking, intimidating, and strict.

## 4 Discussion

The aim of this paper was to explore the association between aspects of the student-supervisor relationship and UK-based doctoral students’ mental health. More specifically, the study investigated the interpersonal style of communication between student and supervisor, as well as the discrepancy between actual and preferred supervisory relationship. It was hypothesized that reported supervisory styles and discrepancy would predict scores in depression, anxiety, and stress. Results supported these two hypotheses, with the uncertain supervisory style significantly predicting mental health scores.

Descriptive statistics indicated that a large proportion of the participants fell in the severe and extremely severe categories in the depression and anxiety sub-scales (22 and 32.6%, respectively). This is in line with previous findings indicating that a large proportion of doctoral students experienced high levels of depression and anxiety both pre-pandemic (e.g., Hazell et al., 2021; Milicev et al., 2021) and during the pandemic (e.g., Sideropoulos et al., 2022). The results of our study were slightly higher in the anxiety sub-scale, with previous studies indicating a smaller proportion of participants in the severe and extremely severe anxiety categories (between 15 and 22%; Hazell et al., 2021; Milicev et al., 2021; Sideropoulos et al., 2022). A possible explanation is that there has been an overall exacerbation of mental health problems as a result of the COVID-19 pandemic that still persists in the post-pandemic period, especially in the case of anxiety-related conditions (see Asmundson and Taylor, 2020; Crawford et al., 2024). Another possible explanation for this difference lies in the use of different tools used to measure anxiety. The aforementioned studies used the Generalized Anxiety Disorder Scale-7 (GAD-7; Spitzer et al., 2006) to measure anxiety. Even though GAD-7 and DASS-21 have been reported to have good convergent validity, this validity is often stronger between GAD-7 and the depression sub-scale of DASS (e.g., Evans et al., 2021; Rutter and Brown, 2016). Therefore, direct comparison between GAD-7 and the anxiety sub-scale of DASS-21 could be problematic. However, a study conducted with doctoral students in Germany during the pandemic (Friedrich et al., 2023) found similar results, where measures of depression and anxiety were significantly higher compared to pre-pandemic reference values using the GAD-7 and Perceived Health Questionnaire (PHQ-9; Kroenke et al., 2001) to measure anxiety and depression, respectively. Additionally, a substantial proportion of our participants (27%) fell in the severe and extremely severe categories of the stress sub-scale, which is in line with previous studies and meta-analyses (e.g., Hazell et al., 2020). Overall, our findings further highlight the recent mental health crisis (Evans et al., 2018) that has been observed in doctoral students in the UK.

In order to answer the first research question, the supervisory styles of the QSDI scale were added as predictors of depression, anxiety, and stress scores. Results indicated that supervisory styles explained a significant proportion of the variance in the sub-scales of depression, anxiety, and stress (26, 12, and 23%, respectively). This is in line with previous literature demonstrating that supervisory relationship is a significant predictor, but not the only important factor influencing doctoral students’ mental health (e.g., Berry et al., 2021, 2023). In fact, a small body of research suggests that, even if doctoral students report a satisfactory supervisory relationship, their wellbeing is influenced by a lack of communication with family and friends and social isolation (e.g., Janta et al., 2014), as well as their personal perceptions of social support (e.g., Gooding et al., 2023). It is therefore possible that doctoral students need both academic and personal support during this difficult journey.

Interestingly, the uncertain supervisory style was the only significant predictor for scores in mental health outcomes, with higher scores in the uncertain supervisory style being linked to higher scores in depression, anxiety, and stress. Looking at the interpersonal model of supervisory relationship, the uncertain supervisory style is found on the low Proximity (Opposition) and low Influence (Submission) side of the model. The style includes indecisiveness and ambiguity during meetings with the supervisor, as well as the supervisor not providing clear directions (Mainhard et al., 2009). This relationship highlights the need for supervisors to be clear, consistent, and decisive during meetings with their doctoral students. Since the supervisory relationship and students’ perception of it is also affected by students’ personal characteristics (Corner et al., 2017), it is possible that students’ own intolerance of uncertainty (IU) plays a role in these results. There are many different definitions of IU (e.g., Carleton, 2016; Freeston et al., 1994). However, a review by Birrell et al. (2011) identified two factors that emerge in research which relies on the most commonly used measure of IU: (1) a need for predictability and a sustained engagement in seeking comfort/certainty, and (2) a dispositional cognitive and physical incapacity in the face of uncertainty. High IU has been associated with various anxiety- and depression-related conditions and is considered to be a transdiagnostic risk factor across a number of psychological disorders (see McEvoy et al., 2019; Morriss et al., 2023). People with high IU were greatly affected during the pandemic, as it was a period of high uncertainty and unpredictability, with research indicating both that people with high IU had the highest rates of depression and anxiety symptoms (e.g., Andrews et al., 2023) and a potential mediating role of IU in the relationship between anxiety and pandemic-related stress (Bredemeier et al., 2023). It is possible that the pandemic affected doctoral students’ IU. This may have further exacerbated their mental health issues while also affecting their ability to cope with ambiguity and uncertainty in their relationship with their supervisor. This is a novel finding which could potentially indicate how the supervisory relationship might influence the mental health issues of doctoral students, but also how IU could be shaping the perception of the supervisory relationship.

Surprisingly, the other supervisory styles were not significant predictors of doctoral students’ levels of depression, anxiety, and stress. This contradicts previous literature that has demonstrated associations between other supervisory styles and mental health issues. Levecque et al. (2017), for example, have suggested strong links between a lack of the leadership supervisory style and mental health issues in doctoral students. One explanation for this difference between our findings and those of other researchers is that the interpersonal model of supervision provided a more complex depiction of supervision in our study, indicating that an uncertain supervisory style is the only set of behaviors related to the mental health and wellbeing of doctoral students. Another likely explanation is that the intercorrelation of the supervisory styles in our study impacted the model’s ability to identify distinct styles that contribute to the prediction of mental health outcomes. Mainhard et al. (2009) highlight that each supervisory style correlates highly with its neighboring and opposite supervisory styles in the model (Figure 1). The uncertain supervisory style is opposite from the leadership style and thus it could be that the high correlation between these two variables resulted in the leadership supervisory style appearing non-significant and the uncertain supervisory style indicating greater significance. Additionally, another possible explanation is that some of the items of the QSDI can be related to more than one supervisory style. For example, the item *“pays attention if I have something to share”* and *“shares my sense of humour”* are found in the understanding style. However, these items could also be included, respectively, in the student responsibility/freedom and helping/friendly styles. This could explain the high correlation of the understanding style with the other supervisory styles. The presence of items which may be related to one or more supervisory styles could make it hard to unambiguously distinguish between the different behaviors and may thus account for our finding that only the uncertain supervisory style had predictive ability.

In order to answer the second research question, three regression models used the Actual-Preferred Supervisory Relationship Discrepancy Scale as a predictor for DASS-21 scores. Results indicated that the scale was a significant predictor of doctoral students’ depression, anxiety, and stress (19, 5.2, and 6.9% of the variance explained, respectively). This study therefore quantitatively supports previous qualitative studies (e.g., Cotterall, 2013) which have suggested that the “fit” of supervision is related to the emotional wellbeing of doctoral students. Moreover, the quick exploratory content analysis indicated that the majority of doctoral students had a positive perception of the supervisory relationship, with few students describing the relationship negatively. However, doctoral students also expressed different expectations and the need for a different approach and relationship from their supervisor. These findings highlight the need for doctoral students and supervisors to communicate the different perceptions and expectations they have from each other and from their relationship (Deuchar, 2008) in order for the relationship to fit the needs of each student (Watts, 2008) and better support the student throughout the doctorate journey.

Even though the study enriched our understanding of the specific aspects of the supervisory relationship that influence the mental health of doctoral students, it is important to acknowledge some of its limitations. First, the study used a cross-sectional design and only provided a small snapshot in time. As supervision might vary over time and along the different research phases of the doctorate degree (Gatfield, 2005), it is important to understand the different types of supervisory styles that are most often perceived as important for the mental health of doctoral students along the doctorate journey. It is possible that throughout their journey, doctoral students will have different needs and expectations from the supervisory relationship. Therefore, following this study, future research could investigate longitudinally how the discrepancy between the actual and preferred supervisory relationship and how the different supervisory styles could be related to mental health outcomes in doctoral students.

A second limitation is that the sample size of the study was relatively small compared to the population of doctoral students in the UK, with an over-representation of females. This may limit the generalizability of the findings. The population of doctoral students in the UK has approximately an equal distribution between males and females (HESA, 2023). Since there is a higher prevalence of mental health problems in females than in males in the UK (McManus et al., 2016), it is possible that the high levels of depression, anxiety, and stress observed are a result of the over-representation of females in the sample. A larger sample size could provide a clearer idea of the prevalence of mental health issues. Furthermore, it could facilitate the exploration of a model where other variables (e.g., student characteristics such as race/ethnicity, socioeconomic background, sexual orientation, learning difficulties) can be either controlled for or explored in relation to mental health problems. Since characteristics such as learning needs may affect students’ perception and experience of supervision (e.g., Collins, 2015), a nuanced understanding of the impact of supervisory style can inform more tailored approaches to doctoral supervision in the future.

Finally, as already mentioned, the interpersonal model of supervision includes supervisory styles which correlate highly with each other (Mainhard et al., 2009). This could potentially be the reason why other supervisory styles, especially the leadership style, did not significantly predict doctoral students’ distress. The inter-correlations might have affected the ability of single styles to show unique contributions. Future studies could look at them separately, using other data analysis methods or using a new supervisory-student interaction questionnaire in order to further explore how the interpersonal style of communication between student and supervisor can predict mental health outcomes.

Despite these limitations, the present study provided new insights in terms of the aspects of the student-supervisor relationship that seem to be related to the psychological wellbeing of doctoral students. Even though it is known that individual, interpersonal, and institutional factors all play a role in the experience and mental health of doctoral students, the current findings provide a clearer understanding that an uncertain supervisory style and a discrepancy between actual and preferred supervisory relationship seem to be most related to students’ negative emotional experiences. One implication of these findings is that, in order to understand the interplay between factors affecting doctoral students’ overall experience, researchers need to explore models of doctoral students’ mental health which include the discrepancy between actual and preferred supervisory relationship, the uncertain supervisory style, and students’ personal characteristics (e.g., the extent to which they can tolerate uncertainty). Another possible implication is that the training of supervisors and university staff in general can be improved through a better understanding of students’ experience and needs along their journey. Supervisors should be equipped with appropriate training and tools on how to support students, identify their unique needs, and provide appropriate mental health guidance and referrals if necessary. Additionally, a risk management approach might be used between supervisors and students as a preventative method to identify the expectations of both student and supervisor from that relationship, as well as the uncertainty that the student can tolerate from that relationship. Exploring the aspects of the doctorate journey and more specifically of the student-supervisor relationship that are important for doctoral students’ mental health and wellbeing contributes to a better understanding of the doctorate experience. It is crucial for universities and research institutions which aim to better support doctoral students along the intellectual and emotionally challenging doctoral journey. Ultimately, this support may enhance students’ mental health, reduce dropout rates, and ensure that students successfully obtain their PhD within reasonable time frames.

## Data Availability

The raw data supporting the conclusions of this article will be made available by the authors, without undue reservation.
